# Natural Antioxidants Beneficial Effects on Anion Exchange through Band 3 Protein in Human Erythrocytes

**DOI:** 10.3390/antiox9010025

**Published:** 2019-12-26

**Authors:** Alessia Remigante, Rossana Morabito, Angela Marino

**Affiliations:** Department of Chemical, Biological, Pharmaceutical and Environmental Sciences, University of Messina, Viale F. Stagno D’Alcontres 31, 98166 Messina, Italy; aremigante@unime.it (A.R.); rmorabito@unime.it (R.M.)

**Keywords:** erythrocytes, band 3 protein, anion exchange, SO_4_^=^, oxidative stress, antioxidants

## Abstract

Band 3 protein (B3p) exchanging Cl^−^ and HCO_3_^−^ through erythrocyte membranes is responsible for acid balance, ion distribution and gas exchange, thus accounting for homeostasis of both erythrocytes and entire organisms. Moreover, since B3p cross links with the cytoskeleton and the proteins underlying the erythrocyte membrane, its function also impacts cell shape and deformability, essential to adaptation of erythrocyte size to capillaries for pulmonary circulation. As growing attention has been directed toward this protein in recent years, the present review was conceived to report the most recent knowledge regarding B3p, with specific regard to its anion exchange capability under in vitro oxidative conditions. Most importantly, the role of natural antioxidants, i.e., curcumin, melatonin and Mg^2+^, in preventing detrimental oxidant effects on B3p is considered.

## 1. Introduction

Oxidative stress is due to an imbalance between reactive oxygen species (ROS) formation and the antioxidant defense systems, initiating pro-oxidant processes. Both radical and non-radical oxygen-based molecules, such as hydroxyl radical (OH•), hydrogen peroxide (H_2_O_2_), singlet oxygen (O_2_), and superoxide (O_2_•) are considered to be ROS [1]. Metabolism basically implies ROS production; however, the organism has an antioxidant defense system, importantly and constantly contributing to keep redox balance at an acceptable level. Among the main endogenous antioxidants, enzymes, such as superoxide dismutase (SOD), catalase (CAT), and glutathione peroxidase (GPx), and non-enzymatic antioxidants, like reduced glutathione (GSH), are able to rapidly neutralize ROS [2]. Antioxidants assumed with the diet, such as vitamin C, vitamin E, carotenoids, minerals (Zn, Mn, Cu, Se, Mg) and polyphenols (flavonoids, phenolic acids), impact the activity of endogenous antioxidants, thus contributing to maintaining redox homeostasis [3]. One of the most critical features in human physiology is redox homeostasis, playing a pivotal role in cellular physiology, as well as in the development of several diseases. Under oxidative stress, the excessive ROS production influences several cell signaling pathways, being a common patho-physiological mechanism which underlies many chronic diseases [4,5,6]. ROS are defined as small highly reactive chemical species, with one or more unpaired electrons, able to oxidize other compounds such as proteins, membrane lipids, nucleic acids and polysaccharides, finally resulting in cell damage.

Early investigations on the human erythrocyte suggested that this cell does not respond to external stimuli and that G proteins, protein kinases, phospholipases and phosphatases contained inside erythrocytes represent non-functional vestiges of signaling pathways active in erythroid precursor cells. However, more recent evidence has demonstrated that human red blood cells are highly responsive to the external environment and that the cell’s machinery related to signaling proteins likely comprises components critically involved in the erythrocyte’s communication with the extracellular medium [7].

It has been shown that oxidative damage reduces survival and rheological properties of circulating red blood cells, affecting their shape, which strictly correlates with Band 3 protein function (B3p) [8,9]. Band 3 protein is importantly involved in maintaining erythrocyte deformability as well as ion balance, and is essential to gas exchange capability, and may account for homeostasis of both erythrocytes and the entire organism under healthy and oxidative stress conditions.

## 2. Role of Band 3 Protein in Erythrocytes

**Figure 1:**
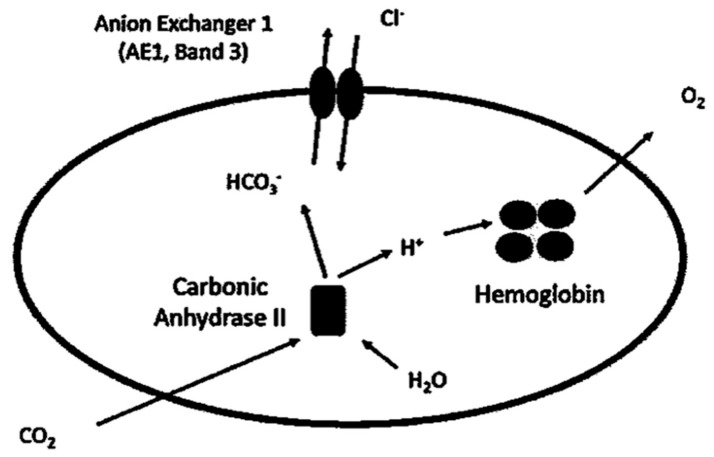
Anion exchange through Band 3 protein [13]. At tissue level, CO_2_ diffuses through erythrocytes membrane and is converted with H_2_O to HCO_3_^−^ and H^+^ by the enzyme carbonic anhydrase II. HCO_3_^−^ is extruded in exchange for Cl^−^, while H^+^ is buffered by hemoglobin. At pulmonary level, the direction of ion exchange through Band 3 protein is reversed (not shown), with HCO_3_^−^ entering the cell in exchange for Cl^−^ and with CO_2_ finally expired by lungs.

Band 3 protein, present in millions of copies on the erythrocyte membrane [10], is the most abundant integral protein, the crystal structure of which was determined in 2015 [11]. The erythrocyte membrane is made of a phospholipid bilayer with integral proteins linked to the cytoskeleton through a protein network underneath the cytoplasmatic side of membrane [12]. Band 3 protein exchanges chloride and bicarbonate (Cl^−^/HCO_3_^−^) anions across the plasma membrane (Figure 1), which is necessary to guarantee efficient respiration [13].

### 2.1. Role in CO_2_ Transport

At tissue level, carbon dioxide (CO_2_) diffusing into erythrocytes is hydrated by intracellular carbonic anhydrase II (CAII), resulting in bicarbonate (HCO_3_^−^) production. This anion is then extruded out of the cell in electroneutral exchange for chloride (Cl^−^) [13,14]. At pulmonary capillary level, the system is reversed: HCO_3_^−^ entering erythrocytes via B3p in exchange for Cl^−^ is converted by CAII to CO_2_, which leaves the cell by permeating through the plasma membrane, to be finally expired by the lungs [15]. Human respiration requires a fast conversion between CO_2_ and HCO_3_^−^, with, on the one hand, CA II facilitating this reversible reaction inside erythrocytes and, on the other hand, B3p providing passage for HCO_3_^−^ across the plasma membrane. These two proteins represent crucial actors of the CO_2_ metabolism. As intracellular H_2_O is needed for CO_2_/HCO_3_^−^ conversion, aquaporin-1 (AQP1), abundantly present in erythrocytes, is considered a part of B3p complexes or involved in CO_2_ transport. With regard to the latter point, the Fluorescence Resonance Energy Transfer (FRET) technique has been useful for identifying the interaction between AQP1 and B3p at 8 nm distance, which falls within the range useful for dipole-dipole interaction. Importantly, interaction between B3p and AQP1 was adaptable to changes in membrane tonicity, which suggests that AQP1 involvement in response to tonicity could be associated with its function in B3p-mediated exchange. For this reason, AQP1 seems to be critically implicated in blood CO_2_ transport and, in turn, respiration [16]. Their primary function is to allow tissue oxygenation and CO_2_ elimination, but erythrocytes, flowing in the blood stream, make tissue oxygenation effective due to their biconcave shape, which corresponds to a greater area available for gas exchange and, notably, lets them adapt to the narrow capillaries.

### 2.2. Role in Erythrocyte Rigidity

Biconcave shape maintaining and erythrocytes size adaptation through capillaries is due to a peculiar plasma membrane arrangement, including structural proteins and cytoskeletal proteins, providing erythrocytes with unique structure.

On the other hand, membrane deformability lets erythrocytes restore their original size when flowing through larger vessels. Therefore, alterations in deformability of erythrocytes may result in changes in microcirculatory blood flow and delivery of oxygen to the tissues. In addition, other factors, including Na^+^/K^+^-ATPase, are also responsible for maintenance of erythrocytes deformability. In particular, Na^+^/K^+^-ATPase, involved in intracellular ionic homeostasis maintenance, consequently affecting cell volume regulation and, in turn, erythrocyte deformability. Reduced deformability of erythrocytes has been shown to have an index of adverse outcomes in the form of cardiovascular diseases, impact on the rheological properties of blood and possibly representing a cardiovascular risk factor [17]. Hence, erythrocyte rigidity represents an important factor affecting oxygen delivery to the tissues, since oxygen-carrying capacity decreases when erythrocytes become more rigid. In this way, increased red blood cell rigidity results in an impaired peripheral perfusion and, finally, tissue oxygenation [18]. As an integral membrane protein, B3p, in addition to gas exchange across the cell membrane, is also involved in erythrocyte mechanical and osmotic properties, such as docking of glycolytic enzymes and maintenance of cell shape [19]. Such functions are mediated by both C-terminal membrane domain for anion exchange and a N-terminal cytosolic domain, which is mainly involved in protein–protein interactions, by coupling plasma membrane to the underlying cytoskeleton, through cysteine –SH groups [20,21,22]. The link between erythrocyte membrane, embedded proteins, namely B3p, and cytoskeletal components is quite complex. According to the model proposed by Kodippili and co-authors [23], a part of B3p is linked to glycophorin A or B, Rh-associated glycoproteins, and other polypeptides bound to cytoskeleton through ankyrin bridge. Another part of B3p is linked to glycophorin C, Rh-associated glycoproteins, GLUT1, actin, protein 4.1 and spectrin, while the remaining part freely diffuses in the lipid bilayer, although interacting with spectrin. Based on this evidence, Reithmeier and co-authors [13] contributed to shedding light on the B3p structure, its arrangement in the lipid bilayer, anion binding and transport. In this regard, B3p functions, in addition to anion exchange and spectrin-actin cytoskeleton anchoring, deal with regulation of glycolytic enzymes, control of erythrocyte lifespan docking for peripheral membrane proteins, such as protein 4.1, protein 4.2, and several phosphatases and kinases. Defects or deficiencies in B3p may lead to a reduced cohesion between cytoskeleton and lipid bilayer, with a consequent loss of membrane surface area typical of hereditary spherocytosis.

Band 3 protein functions, including anion exchange capability, critically depends on a complex net of interactions between lipid bilayer, embedded proteins, hemoglobin and cytoskeleton. With regard to hemoglobin, its link to the erythrocyte membrane contributes to targeting and, in turn, removal of senescent erythrocytes by macrophages. A model has been proposed to explain this process: the link of modified forms of hemoglobin (hemichromes) to plasma membrane affects cytoskeleton integrity, resulting in clustering of B3p. Hemichrome formation, as recently reported by Welbourn and co-authors [24], seems to be involved in storage lesions of blood used for transfusions. Damage deriving from storage may possibly depend on hemichrome binding to the plasma membrane, reduction in the B3p monomer, and increase in B3p degradation products along with a loss of vesicles bearing aggregated hemoglobin, thus resulting in a decreased cell flexibility and a compromised blood flux efficiency.

The exchange of the physiological substrates HCO_3_^−^/Cl^−^ has already been proven by a rate of exchange across the plasma membrane that is so fast (5 × 10^4^ ions/s at 37 °C) that it cannot be easily determined [15]. Nonetheless, the efficiency of B3p can be effectively monitored by measuring the rate constant for the uptake of another anion, SO_4_^=^, slower and, hence, more easily detectable than Cl^−^ or HCO_3_^−^ transport [25]. SO_4_^=^ uptake determination has been recognized as a good tool for monitoring erythrocyte homeostasis [26]. In recent years, B3p anion exchange monitoring has been widely used to assay the effect of different experimental conditions, performed by applying oxidants and toxins in vitro [8,27], or in diseases associated with membrane protein degradation [28,29,30,31]. Consequently, this review, for the first time, collects the most recent knowledge about B3p function, taking into account that red blood cells represent a suitable cell model to evaluate cell response to oxidative conditions, due to their sensitivity to oxidation and simple metabolism, with specific regard to its anion exchange capability. The purpose is to provide a broader and more complete view of the activity of the anion transport mediated by the B3p in human erythrocytes and, more importantly, to report about the beneficial effect of antioxidants on B3p exposed to oxidant conditions in vitro.

## 3. Anion Exchange through Band 3 Protein in Different Oxidative Experimental Conditions and the Role of Antioxidants

Oxidant molecules, transferred by the blood stream, exert their action on the cell membrane with possible effects on transport systems and, in turn, on erythrocyte homeostasis. For this reason, increasing attention has been addressed to ion transport system function and underlying pathways, with the aim of considering them as parameters to verify at which level oxidants act and, possibly, to define novel targets for drug development.

### 3.1. Curcumin

**Figure 2:**
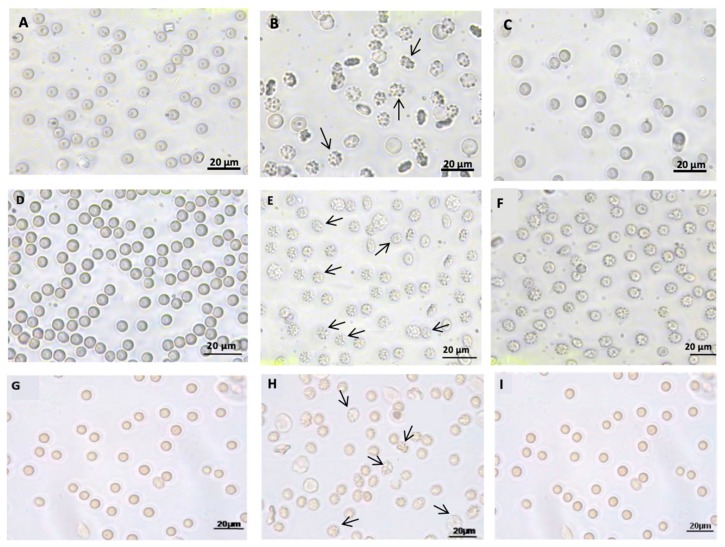
Light microscope observations of untreated human red blood cells [8,34,37,40,41] (**A**,**D**,**G**), or exposed to either pH 6.5 SO_4_^=^ medium (**B**) or to pH 6.5 SO_4_^=^ medium plus 10 μM curcumin [34] (**C**); human erythrocytes exposed to either 300 μM H_2_O_2_ (**E**), or exposed to 10–300 μM H_2_O_2_ (pre-conditioning) [37] (**F**) in SO_4_^=^ medium incubation; human erythrocytes exposed to either 300 µM H_2_O_2_ (**H**), or exposed to 100 Melatonin plus 300 µM H_2_O_2_ [40] (**I**) in SO_4_^=^ medium incubation. 400× magnification. Arrows indicate erythrocytes morphological alterations. Red blood cells shape is irregular due to spines, if compared to the untreated erythrocytes. Pictures are modified from references cited above.

It has been demonstrated that acidification of the external medium (pH 6.5, range not hemolytic) can lead to a significant decrease in anion exchange capability, which is reversed by treatment with Curcumin (Table 1). Curcumin, indian saffron turmeric yellow, is a hydrophobic pigment deriving from the rhizome (turmeric) of *Curcuma longa* herb. It has long been used in medicine and as a food-coloring agent, and its several beneficial effects have already been shown [32,33]. Erythrocytes, when exposed to external medium at different pH values, may exhibit alteration of cytoskeletal and integral membrane proteins, including B3p, resulting in membrane destabilization and ionic imbalance, possibly provoked by oxidative damage (Figure 2). In this latter regard, a significant reduction in anion exchange efficiency has been actually detected, probably due to the acidic medium oxidizing hemoglobin, which produces ROS, with consequent damage to the cell membrane. According to this model, it is clear that perturbations of the external medium may reflect on B3p inefficiency mediated by alterations at level of cytoplasmic proteins [34]. Curcumin protects erythrocyte membranes against oxidative stress by scavenging free radicals, thus acting as an antioxidant molecule [35], similarly to other natural antioxidant compounds. The choice of verifying the efficiency of a specific ion exchanger, such as B3p, was carried out to add more knowledge about the impact of oxidative stress on cell membrane and ion transport system in an anucleate cell.

### 3.2. Hydrogen Peroxide-Induced Oxidative Conditions and the Beneficial Effect of Antioxidants

In addition to the evidence that oxidants provide detrimental effects on membrane transport systems, the damage at the level of B3p may also result from altered interactions with cytoplasmic proteins. To better focus on this aspect, B3p efficiency has been studied when erythrocytes are exposed to hydrogen peroxide (H_2_O_2_). Hydrogen peroxide, commonly used in vitro to model oxidant conditions, is a scarcely reactive non-radical compound easily permeating plasma membranes. It promotes HO• radical formation after binding with transition metals, usually present inside the cell, leading to lipid peroxidation [36]. Therefore, red blood cells, frequently exposed to oxidative conditions [10], may be considered as a good model to study oxidative stress effects despite having potent endogenous antioxidant machineries [8]. It has been shown that H_2_O_2_ induces oxidative stress at not hemolytic concentrations and reduces B3p efficiency, not associated with a reduced cell size [37]. Morphological alterations detected under H_2_O_2_ seem not to be linked to eryptosis, which actually develops at much higher H_2_O_2_ concentrations [38]. In this latter regard, oxidative stress at membrane level may elicit phosphatidylserine (PS) exposure in erythrocytes [39], which implies loss of PS asymmetry, activation of blood coagulation and recognition of red blood cells by macrophages, to remove damaged erythrocytes from circulation.

The reduced efficiency of SO_4_^=^ uptake through B3p, observed under H_2_O_2_-induced oxidative conditions, can be prevented or attenuated by a short-time pre-incubation of red blood cells with low H_2_O_2_ concentrations followed by a stronger oxidative stress (Table 1). Pre-incubation allows red blood cells to adapt to a mild oxidative stress, owing to their higher resistance to oxidants, similarly to what demonstrated on other cell types (Figure 2) [42]. In this case, no antioxidant has been provided from the outside to mitigate the impact of oxidative stress, but the endogenous antioxidant system of erythrocytes, including SOD, CAT, GPx, and Peroxyredoxin 2, is essential to cell survival when oxidative conditions are being applied. Specifically, CAT contributes to ROS neutralization in erythrocytes following oxidative stress induced by H_2_O_2_ [37]. This peculiar response of erythrocytes to oxidative stress is a sort of defense against oxidants and is called preconditioning [37]. This strategy is based on an augmented efficiency of antioxidant enzymes, sustained by low-concentrated oxidants, without the intervention of antioxidant molecules, which represents an impressive homeostatic response of erythrocytes.

Nonetheless, the effect of antioxidants on a validated in vitro model of oxidative stress-induced alterations of B3p has been studied.

### 3.3. Magnesium

In particular, the possible antioxidant effect of Magnesium (Mg^2+^) on erythrocytes following oxidative conditions induced by H_2_O_2_-NEM- Orthovanadate and Diamide has been proved (Table 1) [28,41]. These treatments with oxidants lead to alterations in red blood cells redox state and, moreover, in KCl co-transport activation. Such effects result in the blockage of phosphatase (PTP) activity on B3p tyrosine phosphorylation [43] induced by cell shrinkage. Magnesium, the second most abundant intracellular ion after K^+^, with concentrations ranging between 5 and 30 mM, modulates cell volume regulation, enzymes activity and erythrocytes membrane physical properties [44]. Previous studies on the effect of Mg^2+^ deficiency on membrane function and red blood cell metabolism have been performed in animal models [43,45] and in oxidative stress-related diseases, such as preeclampsia, with hypoxia due to preterm labor [46]. It has already been reported that, in hamsters, magnesium deficiency increases red blood cells’ susceptibility to injury due to free radicals and, in rats, provokes a significant reduction in red blood cells GSH, restored by administration of d-propanolol or vitamin E [47,48]. Hence, Mg^2+^ has been chosen as a good candidate to study its possible beneficial effect in protecting B3p anion exchange capability in case of oxidative damage.

Among the most interesting outcomes, magnesium pre-exposure has been shown to prevent the reduced efficiency of SO_4_^=^ uptake through B3p, in both normal and G6PDH-deficient red blood cells, characterized by reduced GSH production and greater sulfhydryl-group (–SH groups) oxidation in in vitro models [28,41]. Specifically, the evidence that oxidative stress decreases the efficiency of anion transport could be due to either structural changes of B3p by SH-groups oxidation or to cell shrinkage after increased K^+^ efflux [28]. In this regard, Mg^2+^ exerts its effect on KCC-mediated cell shrinkage observed under oxidative stress. To better focus on the possible antioxidant effect of Mg^2+^, phosphorylation of Tyrosine and Syk kinases expression levels have been measured in normal erythrocytes following oxidative stress induced by H_2_O_2_ and NEM [41]. It is already known that human erythrocytes provide a metabolic response to oxidative conditions in order to maximize the production of NADPH, to restore GSH content and Thioredoxin, and to activate Syk kinases, responsible for phosphorylation of Tyrosines associated with B3p oxidation [43]. The hyperphosphorylation of B3p has been described in several prooxidant hemolytic disorders [49], and it has been shown that oxidized B3p is selectively phosphorylated [50] by Syk kinase, which is responsible for Tyr 8 and Tyr 21 phosphorylation [43,51,52]. Magnesium beneficial effect is not mediated by phosphorylative pathways, but the metal would prevent alterations and protects erythrocyte homeostasis via membrane organization, cross linking between B3p and cytoplasmic proteins, thus impairing Syk docking. Certainly, the rate constant restoration due to pre-exposure to Mg^2+^ also depends on GSH and –SH group protection, suggesting a role of endogenous antioxidant system stabilized by Mg^2+^ [28,41,49].

### 3.4. Melatonin

Melatonin (Mel) is an antioxidant that has been used both in vitro and in vivo on animal models or in clinics to counteract oxidative stress-associated pathologies. Melatonin, a neurohormone derived from tryptophan and predominantly produced by the pineal gland of vertebrates [53], is a multifunctional and evolutionarily conserved molecule acting as a powerful antioxidant fighting against oxidative stress [54,55,56]. In vivo studies have already demonstrated that Mel displays antioxidant properties even greater than those of vitamin E, vitamin C, and α-carotene [54,55]. Additionally, Mel can promote the function of various enzymatic antioxidants, i.e., GPx SOD, GR, and CAT, as well as GSH synthesis, increasing intracellular GSH content [55,57]. In a recent study, a correlation between Mel effect, oxidative stress, and B3p anion exchange capability was shown for the first time. In particular, the authors reported that pre-treatment of red blood cells with Mel impairs the reduction in rate constant for SO_4_^=^ uptake (Table 1) and the reduction in B3p expression levels due to H_2_O_2_. Since H_2_O_2_-induced oxidative stress on red blood cells involves neither lipid peroxidation nor oxidation of membrane –SH groups nor methaemoglobin (MetHb) formation [37], the beneficial effect of Mel in protecting B3p should not depend on these mechanisms. Hence, to shed more light on the effect of this molecule, Mel antioxidant power has been assayed in the absence of CAT activity. In such experimental conditions, Mel’s efficacy in impairing the reduction of the rate constant for SO_4_^=^ uptake due to oxidative conditions and in protecting B3p expression levels was demonstrated [40]. Melatonin also exerts its effect by delaying membrane protein degradation and precipitation of hemin on red blood cells membrane. This could probably explain the preventative effect of Mel against the reduction in B3p expression levels observed after H_2_O_2_, thus corroborating the hypothesis that the beneficial action of Mel is exerted at the membrane level (Figure 2) [58,59].

## 4. Conclusions

Erythrocytes are reasonably employed as models for studies on cellular homeostasis, being continuously threatened by oxidative events associated with high ROS levels. The present review offers more knowledge about the effect of oxidative stress on exchange through B3p under different in vitro experimental conditions, with the double purpose of not only considering B3p efficiency as a good tool for monitoring erythrocyte homeostasis, but also of making it eligible as a target for antioxidants to protect erythrocytes homeostasis. In this context, what has been performed in vitro to study the impact of oxidative stress along with the beneficial effect of antioxidants on B3p could contribute to propose an effective use of antioxidant compounds, possibly introduced by diet, to prevent detrimental effects of oxidative conditions, namely at erythrocytes level. Hence, antioxidant supplementation can be reasonably considered as a tool for improving the endogenous antioxidant defense and to fight against free radicals.

## Figures and Tables

**Table 1:** Rate constant (min^−1^) for SO_4_^=^ uptake.

Condition	Rate Constant (min^−1^)	Time (min)	*n*
Human erythrocytes (ctr) [8,34,37,40,41]	0.066 ± 0.001	18	18
Medium pH 6.5 [34]	0.035 ± 0.001	29	6
300 μM H_2_O_2_ [37]	0.032 ± 0.001	31	6
600 μM H_2_O_2_ [37]	0.030 ± 0.001	33	3
1 mM Diamide [28]	0.029 ± 0.002	32	5
0.1 mM Orthovanadate [28]	0.031 ± 0.001	34	5
0.5 mM NEM [41]	0.030 ± 0.001	33	5
1 mM NEM [41]	0.033 ± 0.003	30	5
2 mM NEM [41]	0.023 ± 0.002	43	7
10 μM Curcumin in Medium pH 6.5 [34]	0.048 ± 0.001	20	6
10 mM Mg^2^+ 300 μM H_2_O_2_ [41]	0.058 ± 0.005	17	5
10 mM Mg^2^+ 600 μM H_2_O_2_ [41]	0.057 ± 0.001	17	5
10 mM Mg^2^+ 0.5 mM NEM [41]	0.060 ± 0.002	16	6
10 mM Mg^2^+ 1 mM NEM [41]	0.056 ± 0.002	18	6
10 mM Mg^2^+ 2 mM NEM [41]	0.055 ± 0.002	18	6
100 μM Melatonin + 300 μM H_2_O_2_ [40]	0.078 ± 0.001	13.5	10
10 μM H_2_O_2_ (Preconditioning) + 300 μM H_2_O_2_ [37]	0.051 ± 0.001	19	4
